# An anatomical perspective on clinicopathological characteristics and treatment outcomes of dorsal and ventrolateral tongue leukoplakia after carbon dioxide laser surgery

**DOI:** 10.1186/s12903-021-01403-8

**Published:** 2021-01-28

**Authors:** Shih-Wei Yang, Yun-Shien Lee, Liang-Che Chang, Cheng-Han Yang, Cheng-Ming Luo

**Affiliations:** 1 Department of Otolaryngology Head and Neck Surgery, Chang Gung Memorial Hospital, Keelung. No. 222, Mai Chin Road, Keelung, 204 Taiwan, ROC; 2 College of Medicine, Chang Gung University, Taoyuan, Taiwan, ROC; 3 New Taipei Municipal Tucheng Hospital, New Taipei City, Taiwan, ROC; 4 Genomic Medicine Research Core Laboratory, Chang Gung Memorial Hospital, Tao-Yuan, Taiwan, ROC; 5 Department of Biotechnology, Ming Chuan University, Tao-Yuan, Taiwan, ROC; 6 Department of Pathology, Chang Gung Memorial Hospital, Keelung, Taiwan, ROC

**Keywords:** Leukoplakia, Tongue, Dorsal, Ventrolateral, Malignant transformation, Recurrence, Carbon dioxide laser, Carcinoma

## Abstract

**Background:**

The tongue has been identified as a high-risk site for malignant transformation of oral leukoplakia. The purpose of this study was to investigate the clinicopathological characteristics and treatment outcomes of the dorsal and ventrolateral tongue leukoplakia.

**Methods:**

Demographic data and pathological results of patients who underwent carbon dioxide laser surgery for tongue leukoplakia from 2002 to 2019 were retrospectively reviewed and analyzed statistically.

**Results:**

Of the 111 patients enrolled, 80 were males and 31 females, with a mean age of 51.86 ± 11.84 years. The follow-up time was 3.74 ± 4.19 years. Fifteen patients had a postoperative recurrence (13.51%). Four (3.6%) patients developed malignant transformation. Annual transformation rate was 4.03%. There were no differences in the time to develop carcinoma (3.19 ± 1.94 vs. 3.51 ± 2.12 years, *P* = 0.83), overall cumulative malignant transformation rates (7.41% vs. 2.25%, *P* = 0.12), and annual transformation rates (2.32% vs. 0.64%, *P* = 0.099). The prevalence of the ventrolateral tongue leukoplakia was higher than that of the dorsal tongue leukoplakia (*P* < 0.001). The results of multivariate logistic regression analysis showed that the degree of pathology was the only independent prognostic factor related to postoperative malignant transformation (*P* = 0.045).

**Conclusions:**

Dorsal tongue leukoplakia is not as frequently encountered clinically as ventrolateral tongue leukoplakia. The response of the dorsal tongue and ventrolateral tongue leukoplakia to laser therapy of are comparable in postoperative recurrence and postoperative malignant transformation. Clinicians should take a more aggressive attitude toward oral tongue leukoplakia with higher grade of dysplasia.

## Background

Approximately 300,000 new cases of oral squamous cell carcinoma occur annually and the treatment outcomes and survival are highly stage-dependent [1, 2]. Although oral cancers can occur de novo, large number of oral cancers are derived from oral potentially malignant disorders. Among the disorders, oral leukoplakia (OL) is the most commonly seen [3]. OL on the tongue and floor of the mouth was thought to be the locations with a higher risk of malignant change [4–6]. The tongue occupies the major portion of the floor of the mouth and is divided by the circumvallate papillae into the anterior two-thirds (mobile tongue or body) and the base of the tongue [7, 8]. The dorsal tongue and ventrolateral tongue are different parts of the mobile tongue, and the morphological outlook, development, structure, function, and histology are different [9, 10]. Among the studies on tongue leukoplakia, most of which focus on the lateral and ventral tongue and research on the dorsal tongue leukoplakia is limited thus far, so the aim of this study was to investigate and compare the dorsal tongue and ventrolateral tongue leukoplakia from the perspectives of histopathology, clinical characteristics, and treatment responses.

## Methods

This study was approved by the Chang Gung Medical Foundation Institutional Review Board (License No. 201901384B0). Medical records of patients with oral tongue leukoplakia who underwent carbon dioxide laser (CO_2_ laser) excision at the Department of Otolaryngology from September 2002 to October 2019 were retrospectively reviewed. All methods were carried out in accordance with the relevant guidelines and regulations (Declaration of Helsinki).

All the patients underwent a thorough oral cavity examination by an otolaryngology specialist. Anesthesia was composed of lidocaine (1%, 50 mg in 5 mL) and epinephrine (1 mg/1 mL) and the ratio was 20:0.1. Usually 5–10 mL of the anesthesia was injected around the area of leukoplakia. The dosage needed was dependent on the area of the leukoplakia. Transoral laser excision was then performed as previously described [11–13]. The laser surgeries were performed by a doctor (S.-W.Y.) under local anesthesia and the patients remained conscious during the surgical procedures. All specimens were sent for pathological examination and the pathological diagnosis was confirmed and agreed upon by two different pathologists. A binary grading system proposed by the WHO was adopted to diagnose the pathology [14]. Before surgery, the types of leukoplakia of every patient, including homogeneous and non-homogeneous [5, 15] were first evaluated and photographed by the author (S.-W.Y.). The images were later reviewed by two specialists in otolaryngology and a consensus on the clinical appearances was reached. The inclusion criteria consisted of a clinical diagnosis of leukoplakia on the mobile tongue with or without leukoplakia on the other parts of the oral cavity mucosa, patients’ age older than 20 years, and treatment with CO_2_ laser. Other types of OPMDs except leukoplakia (such as submucous fibrosis, lichen planus, erythroplakia, and so on.), previous treatment of OL at other medical facilities, no agreeable pathological diagnosis made, initial pathological diagnoses being carcinoma or malignancies, papilloma with a gross papillary appearance, obvious ulceration, overt carcinoma on inspection, or treatment with laser vaporization were excluded. The history of betel nut or areca chewing, alcohol drinking, and cigarette smoking were obtained by detailed questioning of the patient at the patients' first visit to the outpatient department [13]. The surface area of the leukoplakia was measured on the excised specimen immediately after the laser operation.

Postoperative recurrence is defined as OL regrowth on the same site after confirmation of no evidence of any OL lesion for a definite period [16]. If a lesion of tongue leukoplakia occurred at a different location from the previous surgically treated site, it was defined as a second lesion instead of recurrence. If a patient had one or more than one lesion of leukoplakia exclusively on the mobile tongue, the situation was defined as “single.” “Multifocal condition” described leukoplakia involving the oral mucosa in addition to the mobile tongue. The highest degree of pathology and the most severe form of clinical presentation of tongue leukoplakia were documented for analysis and statistical calculation on a per capita basis.

The postoperative follow-up course was uneventful. All the patients were able to come back to the office as scheduled without any major morbidities, including wound infections causing systemic septicemia, massive hemorrhage, paresthesia, impaired mobility of the tongue, change of taste sensations, and so forth.

### Statistical analysis

Results were descriptively presented, with factors related to postoperative recurrence and malignant transformation of tongue leukoplakia. For univariate analysis, Fisher’s exact test, and one-way analysis of variance between groups were performed. Survival analyses were performed using Kaplan–Meier curves with log-rank tests (for factors with two groups of subjects) and logistic regression model (for continuous variables such as body mass index, area of leukoplakia, or combined calculations of factors). Odds ratios (ORs), hazard ratios (HRs), and 95% confidence intervals (CIs) were calculated using a 2-tailed test of significance (*P* < 0.05) for each factor. We followed the following parameters: (1) when the 95% CI did not include 1.0, the resulting OR (or HR) of the risk factor was statistically significant; (2) if the value of the OR (or HR) was greater than 1.0, the risk was increased, and (3) if the value was less than 1.0, the risk was reduced or protective. Prevalence was compared using the incidence rate ratio [17]. Fisher’s exact tests were calculated using the MATLAB version R2015a (Mathworks Inc., Natick, Mass., USA).

## Results

**Fig. 1:**
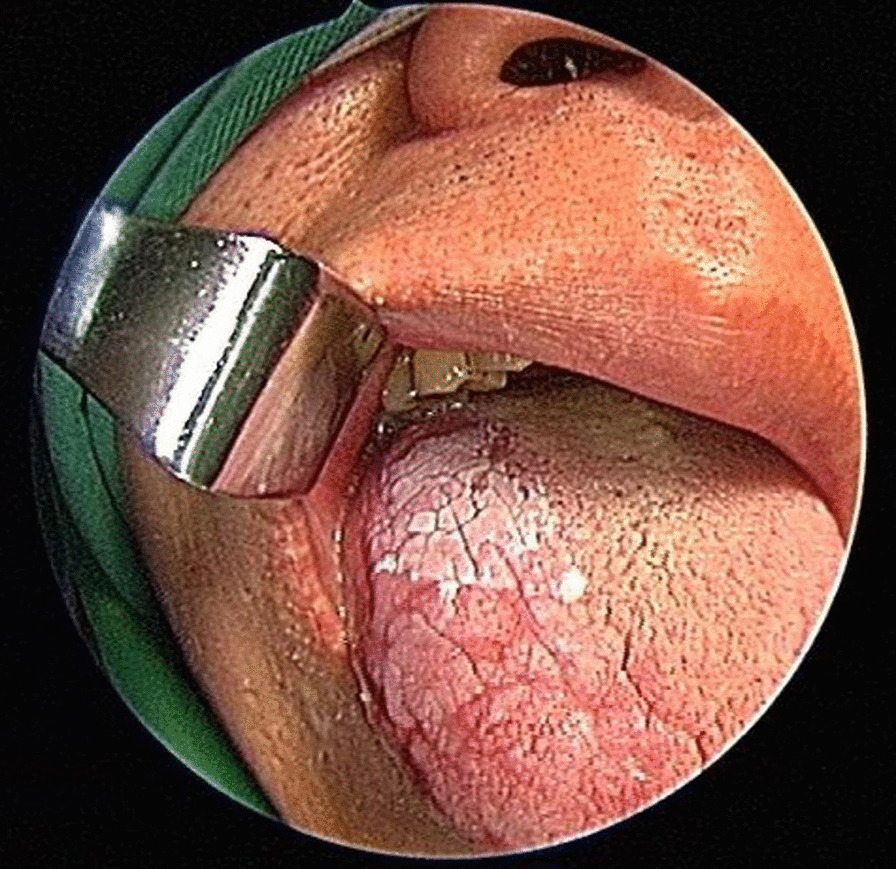
Right dorsal tongue non-homogeneous leukoplakia of a 48-year-old male patient

**Fig. 2:**
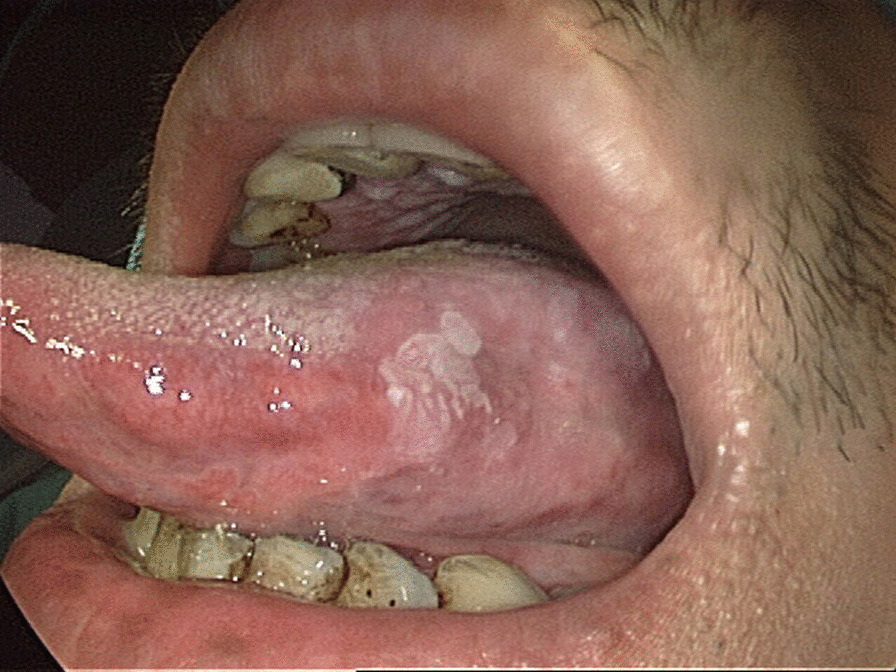
Left lateral tongue non-homogeneous leukoplakia of a 38-year-old male patient

**Fig. 3:**
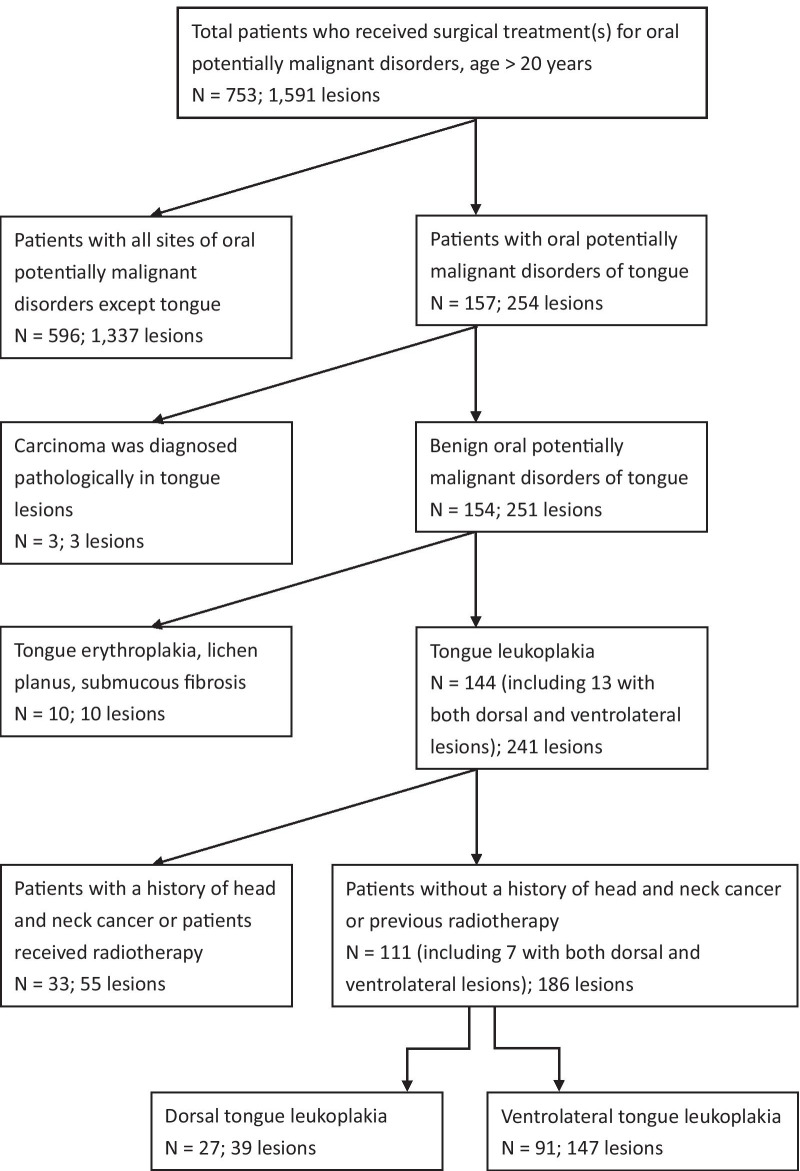
Algorithm for identifying study cohorts

Seven hundred and fifty-three patients with 1591 OPMD lesions who underwent CO_2_ laser surgery for tongue leukoplakia at the department from 2002 to 2019 were recruited. Excluding patients with OPMDs occurring not on the oral tongue, clinical tongue OPMDs other than leukoplakia, an initial diagnosis of carcinoma, and patients with a history of head and neck cancer or radiotherapy in the head and neck regions, 111 patients with 186 lesions of tongue leukoplakia were enrolled, including 27 patients with 39 lesions of dorsal tongue leukoplakia and 91 patients with 147 lesions of ventrolateral tongue leukoplakia (Figs. 1, 2). Among the 111, 7 patients had both dorsal tongue and ventrolateral tongue leukoplakia (Fig. 3).

Among the 111 patients, 80 were males and 31 were females, whose ages ranged from 25 to 78 years with an average of 51.86 years. The average follow-up time was 44.94 ± 50.31 months. Multiple lesions could occur on both the tongue and other locations of the oral cavity in some of the enrolled patients. Different clinical presentations, such as homogeneous and non-homogeneous leukoplakia, could occur at different sites of the oral cavity or on the same site in cases with postoperative recurrence. Histopathological examinations of different severity also possibly happened in the different locations of lesions in the same patient. It was not possible to correlate every patient with a single morphological appearance or pathological examination unless the patient had only one lesion. Therefore, the most severe form of morphology and the highest degree of pathological severity of tongue leukoplakia were documented on a per capita basis. In this series, 60 out of 111 patients (54.05%) had multifocal lesions, or OL on other sites in addition to tongue leukoplakia, including buccal leukoplakia in 54 patients, retromolar leukoplakia in 13, gum leukoplakia in 6, labial leukoplakia in 3, the floor of mouth leukoplakia in 1, and palate leukoplakia in 1. Among the 61 patients (45.95%) with only tongue leukoplakia, 51 patients had solitary tongue leukoplakia during the cohort study. Eighty-three patients had homogeneous tongue leukoplakia and 28 had non-homogeneous tongue leukoplakia (74.77% vs. 25.23%). The number of cases of squamous hyperplasia, mild dysplasia, moderate dysplasia, and severe dysplasia/carcinoma in situ (CIS) was 34, 51, 16, and 10, respectively. If a binary classification was adopted [14], the number of low-risk lesions (85 cases, including squamous hyperplasia and mild dysplasia) surpassed that of the high-risk lesions (26 cases, including moderate dysplasia and severe dysplasia/CIS). The average area of tongue leukoplakia was 1.48 ± 1.53 cm^2^. Fifteen patients (13.51%) had a postoperative recurrence. Four patients (3.6%) had postoperative malignant transformation of tongue leukoplakia. The mean time for the development of recurrence was 3.35 ± 1.67 years. The annual recurrence rate was 4.03%. The average time for malignant transformation was 3.35 ± 1.67 years. The annual transformation rate (ATR) was 2.79%. The demographic and clinicopathological data are shown in Table 1. All 4 patients with tongue leukoplakia transformed into OSCC were males and their ages ranged from 42 to 62 years. The locations of malignant transformation of oral tongue leukoplakia were equally distributed on the dorsal and ventrolateral tongue (2 cases in each location). The pathology of oral tongue leukoplakia included mild, moderate, and severe dysplasia in 1, 2, and 1 cases, respectively. One of the 4 cases had a postoperative recurrence of tongue leukoplakia. A summary of the demographic data and clinicopathological information of the 4 patients with tongue leukoplakia treated by carbon dioxide laser which transformed into carcinoma was shown in Table 2.

**Table 1:** Characteristics of patients who received laser surgery for tongue

	Case no.	%
*leukoplakia (n* = *111)*		
Gender		
Female	31	27.93
Male	80	72.07
Age (mean ± standard deviation: 51.86 ± 11.84 years old)		
< 65	95	85.59
≥ 65	16	14.41
Alcohol drinking		
No	74	66.67
Ex-drinker	26	23.42
Current drinker	11	9.91
Smoking		
No	34	30.63
Ex-smoker	30	27.03
Current smoker	47	42.34
Betel quid chewing		
No	61	54.95
Ex-chewer	43	38.74
Current chewer	7	6.31
Diabetes mellitus^a^		
No	87	79.82
Yes	22	20.18
Metformin taken^b^		
No	89	83.18
Yes	18	16.82
Occurrence of leukoplakia in addition to tongue^c^		
No (single)	51	45.95
Yes (multi-focal)	60	54.05
Candida infection^d^		
No	103	92.79
Yes	8	7.21
Subsites of tongue leukoplakia^d^		
Dorsal tongue mucosa	27	22.88
Ventrolateral tongue mucosa	91	77.12
Morphological outlooks		
Homogeneous	83	74.77
Non-homogeneous	28	25.23
Histopathological diagnosis		
Squamous hyperplasia	34	30.63
Mild dysplasia	51	45.95
Moderate dysplasia	16	14.41
Severe dysplasia / carcinoma in situ	10	9.01
Postoperative recurrence		
No	96	86.49
Yes	15	13.51
Postoperative malignant transformation		
No	107	96.40
Yes	4	3.60
Body mass index^e^	26.42 ± 4.71	
Area (cm^2^) of the lesion(s)^f^	1.48 ± 1.53	
Cumulative malignant transformation rate	3.60%	
Duration of follow-up (year)	3.74 ± 4.19	
Time to develop recurrence (year)	4.84 ± 4.36	
Time to develop carcinoma (year)	3.35 ± 1.67	
Annual recurrence rate^g^	2.79%	
Annual transformation rate^g^	1.08%	

^a^Two pieces of missing data in the group of diabetes mellitus (n = 109)

^b^Four pieces of missing data in the group of metformin taken (n = 107)

^c^If a patient has other sites of oral leukoplakia in addition to tongue, the patient will be categorized as "Yes"

^d^Seven patients had both dorsal and ventrolateral tongue leukoplakia

^e^Four pieces of missing data if the group of body mass index (n = 107)

^f^If the patient has more than 1 site of tongue leukoplakia, the area is the sum of all tongue leukoplakia lesions

^g^The annual recurrence rate and annual transformation rate is calculated by the recurrence rate and malignant transformation rate divided by the average time of development of recurrence or carcinoma (year)

**Table 2:** The demographic data and clinicopathological information of the patients whose tongue leukoplakia treated by carbon dioxide laser transformed into carcinoma

Gender	Age	Smoking	Drinking	Betel	Oral tongue leukoplakia							Oral squamous cell carcinoma	
					Location	Multifocus	Area (mm^2^)	*Candida* infection	Pathology	Recurrence	Follow-up time (years)	TNM stage (AJCC, 8th)	Time to develop carcinoma (years)
Male	42.0	Smoker	No	Ex-chewer	Dorsal tongue, right	Yes	5.1	No	Mild dysplasia	No	5.32	T1N0M0, stage I	4.56
Male	62.0	Ex-smoker	Ex-drinker	Ex-chewer	Dorsal tongue, left	No	0.15	No	Severe dysplasia	No	4.45	T3N1M0, stage III	1.82
Male	53.0	No	No	No	Ventrolateral tongue, right	Yes	3	No	Moderate dysplasia	Yes	3.64	T1N0M0, stage I	2.01
Male	55.0	Smoker	No	Ex-chewer	Ventrolateral tongue, left	No	1.8	No	Moderate dysplasia	No	6.31	T1N0M0, stage I	5.01

**Fig. 4:**
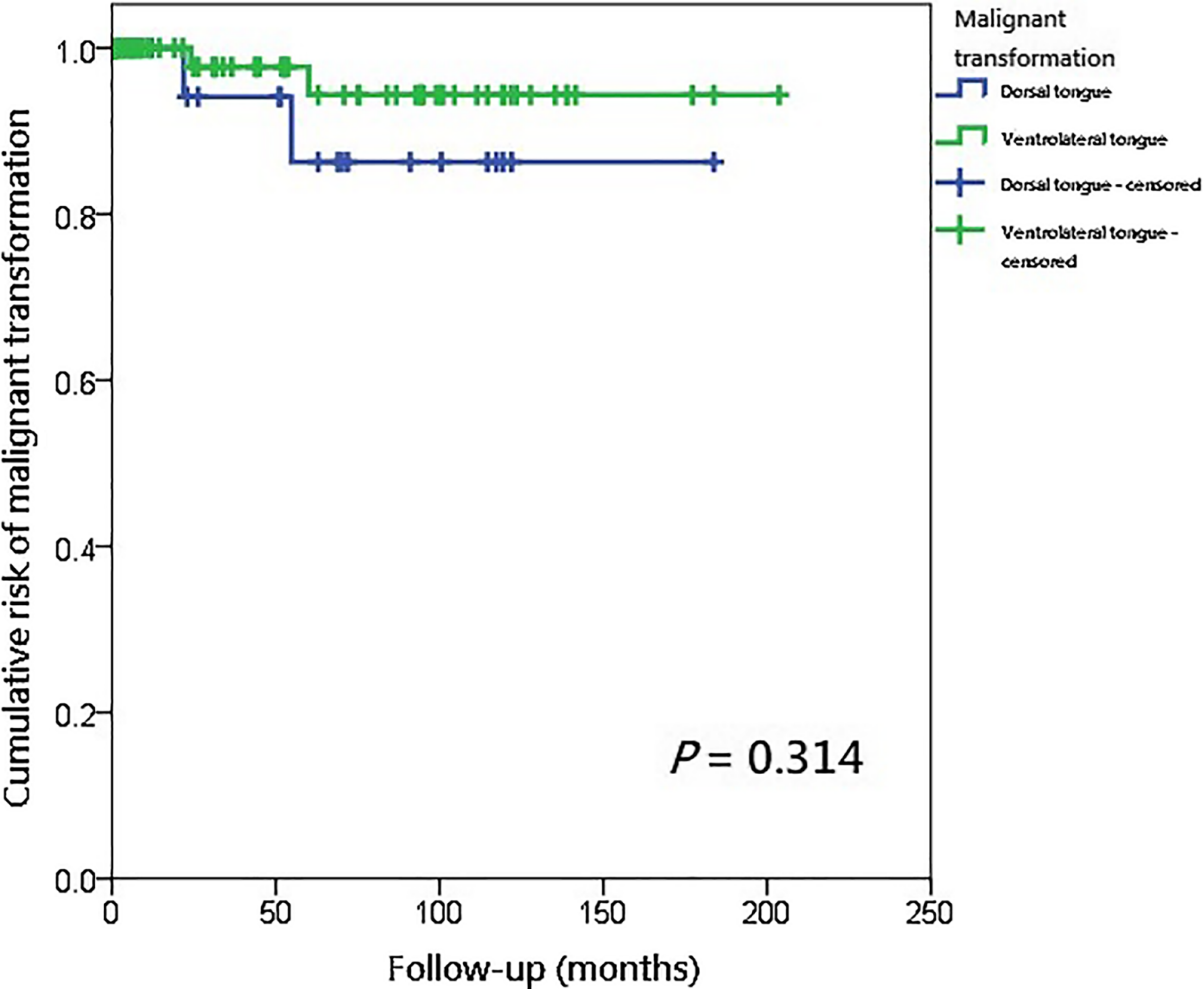
Kaplan–Meier analysis with a log rank test of annual transformation rate in patients whose leukoplakia was on the dorsal tongue (n = 27) (blue line) versus ventrolateral tongue (n = 91) (green line)

In the comparison of the clinicopathological characteristics and treatment outcomes of the dorsal tongue and ventrolateral tongue leukoplakia, only 1 factor (prevalence of the lesions, *P* < 0.001, Table 3) was significantly different between the two sites of tongue leukoplakia. There were no statistical differences in postoperative recurrence, malignant transformation, cumulative malignant transformation rate, and annual transformation rate between the dorsal tongue and ventrolateral tongue leukoplakia (Table 3). The postoperative malignant transformation of the dorsal tongue and ventrolateral tongue leukoplakia was analyzed using the Kaplan–Meier survival analysis model and a log-rank test, which showed no significant difference (*P* = 0.314, Fig. 4). The results of multivariate logistic regression analysis showed that the degree of pathology was the only independent prognostic factor related to the postoperative malignant transformation (*P* = 0.045, Table 4).

**Table 3:** The comparison of clinicopathological characteristics and treatment outcomes in patients with dorsal and ventrolateral tongue leukoplakia (n = 111)

	Dorsal tongue leukoplakia (n = 27)^a^	Ventrolateral tongue leukoplakia (n = 91)^a^	Odds ratio	*P* value
*Clinicopathological data*				
Gender				0.81
Female	7	27	1	
Male	20	64	0.83 (0.31–2.19)	
Age				0.36
< 65	25	77	1	
≥ 65	2	14	2.27 (0.48–10.69)	
Body mass index	24.83 ± 4.34	26.63 ± 4.88^b^	1.10 (0.99–1.22)	0.091
Alcohol drinking				0.17
No	15	64	1	
Ex-drinkYes (ex-drinker or current drinker)	12	27	1.89 (0.78–4.58)	
Smoking				0.38
No	6	30	1	
Ex-smoker	10	23	0.46 (0.15–1.45)	
Current smoker	11	38	0.69 (0.23–2.08)	
Betel quid chewing				0.27
No	12	53	1	
Ex-chewer	14	31	0.50 (0.21–1.21)	
Current chewer	1	7	1.58 (0.18–14.12)	
Diabetes mellitus^c^				0.78
No	21	72	1	
Yes	4	19	1.39 (0.42–4.52)	
Metformin taken^d^				0.76
No	22	73	1	
Yes	3	16	1.61 (0.43–6.03)	
Prevalence (%)^e^	3.39% [49 in (1591–147)]	9.53% [147 in (1591–49)]	2.81 (2.03–3.88)	**< 0.001**
Occurrence of leukoplakia in addition to tongue^c^				0.39
No (single)	10	43	1	
Yes (multi-focal)	17	48	0.66 (0.27–1.59)	
Candida infection^g^				0.68
No	26	84	1	
Yes	1	7	2.17 (0.25–18.43)	
Morphological outlooks				1
Homogeneous	20	66	1	
Non-homogeneous	7	25	1.08 (0.41–2.87)	
Area (cm^2^) of the lesion(s)^h^	1.47 ± 1.73	1.48 ± 1.47	1.01 (0.76–1.34)	0.96
Pathology				0.78
Hyperplasia	8	27	1	
Mild dysplasia	15	42	0.69 (0.27–1.77)	
Moderate dysplasia	3	13	0.93 (0.26–3.27)	
Severe dysplasia / carcinoma in situ	1	9	0.55 (0.17–1.72)	
*Treatment outcomes*				
Postoperative recurrence				1
No	24	78	1	
Yes	3	13	1.33 (0.35–5.07)	
Postoperative malignant transformation				0.22
No	25	89	1	
Yes	2	2	0.28 (0.04–2.10)	
Cumulative malignant transformation rate	7.41%	2.25%	0.30 (0.04–2.16)	0.12
Time to develop malignant transformation (year)	3.19 ± 1.94	3.51 ± 2.12	1.16 (0.30–4.55)	0.83
Annual transformation rate^i^	2.32%	0.64%	0.28 (0.03–1.96)	0.099
Duration of follow-up (year)	4.41 ± 4.16	3.73 ± 4.35	0.97 (0.88–1.06)	0.47

The bold values stand for *P* < 0.05

^a^There were 7 patients who had leukoplakia both on the dorsal and ventrolateral tongues

^b^Four pieces of missing data in the group of body mass index of patients with ventrolateral tongue leukoplakia (n = 87)

^c^Two pieces of missing data in the group of diabetes mellitus (n = 116)

^d^Four pieces of missing data in the group of metformin taken (n = 114)

^e^The prevalence of lesions is calculated by the number of tongue leukoplakia divided by that of all oral cavity leukoplakia in this study. There were totally 54 dorsal and 187 ventrolateral tongue leukoplakia lesions, including recurrent lesions

^f^If a patient has other sites of oral leukoplakia in addition to the tongue, the patient will be categorized as "Yes"

^g^The diagnosis of candida infection is made by pathology

^h^If the patient has more than 1 site of tongue leukoplakia, the area is the sum of all tongue leukoplakia lesions

^i^The annual transformation rate is calculated by the malignant transformation rate divided by the average time of development of carcinoma (year)

**Table 4:** Multivariate analysis of postoperative malignant transformation of patients with tongue leukoplakia (n = 111)

	Postoperative malignant transformation	
	Odds ratio (Confidence interval 95%)	*P* value
Age	1.02 (0.92–1.14)	0.693
Location of leukoplakia (dorsal vs. ventrolateral)	8.08 (0.70–93.47)	0.094
Occurrence of leukoplakia in addition to tongue	1.24 (0.12–12.46)	0.86
Area (cm^2^) of the lesion(s)	1.53 (0.85–2.75)	0.16
Pathology	**4.58 (1.04**–**20.24)**	**0.045**
Postoperative recurrence	0.74 (0.049–11.18)	0.83
Constant	NA	0.032

The bold values stand for *P* < 0.05

*NA* not available

## Discussion

Limited information exists on the clinicopathological characteristics and treatment outcomes specific to leukoplakia of the dorsum of the tongue. Dorsal tongue leukoplakia has not been specifically addressed in the post research and was usually incorporated into the OL of all parts of oral cavity. Therefore, the present study is the first to analyze the clinicopathological features and therapeutic effects of CO_2_ laser on the dorsal tongue leukoplakia. In addition, we made a comparison between the dorsal and ventrolateral tongue leukoplakia to investigate if there were differences, which is also addressed for the first time in the literature. Our series showed that there were no significant differences of clinicopathological features between the dorsal and ventrolateral tongue leukoplakia, including gender, age, body mass index, history of head and neck cancer, alcohol drinking, cigarette smoking, betel quid chewing, diabetes mellitus, taking metformin, concomitant occurrence of leukoplakia on the other parts of the oral mucosa, *Candida* infection, area of the lesions, and pathology, except prevalence (*P* < 0.001). Twenty-seven patients with 39 lesions (3.59%, Fig. 3) of dorsal tongue leukoplakia were enrolled in our series over a period of 17 years. Compared with the cases of ventrolateral tongue leukoplakia (12.08%, 91 patients with 147 lesions, Fig. 3), the prevalence of the dorsal tongue leukoplakia was significantly less than that of the ventrolateral leukoplakia (*P* < 0.001, odds ratio 2.81, 95% CI 2.03–3.88, Table 3). In other words, the dorsal tongue leukoplakia is not as frequently encountered clinically as the ventrolateral leukoplakia. A similar finding was also noted in another study, where only 3 cases of dorsal tongue leukoplakia were found among 38 lesions [18]. The same phenomenon seems to exist in patients with squamous cell carcinoma of the tongue. Carcinoma of the dorsum of the tongue occurs in 3‒5% of all cases of tongue carcinoma, which is far less frequently seen than ventrolateral tongue carcinoma [9, 19, 20]. The prognosis of the dorsal tongue carcinoma was worse in a study carried out in Hong Kong in 65 tongue cancer patients treated by surgery. The 5-year survival rate of patients with the ventrolateral tongue cancer was 51%, whereas the 5-year survival rate for the dorsal tongue cancer was 0% [21]. On the contrary, as for the treatment outcomes of mobile tongue leukoplakia in the present study, the prognosis in these two sites was not significantly different, including the postoperative recurrence rate (11.11% vs. 14.29%, *P* = 1.0), cumulative malignant transformation rate (7.41% vs. 2.25%, *P* = 0.22), and ATR (2.32% vs. 0.64%, *P* = 0.099, Table 3).

In a meta-analysis of 24 studies of OL treated with CO_2_ laser, the overall cumulative malignant transformation rate was 4.5% [22]. Another systematic review of 24 articles about malignant development of carcinoma of OL demonstrated that the estimated overall cumulative malignant transformation rate was 3.5% [23]. In the present study, the overall cumulative malignant transformation rate of the oral tongue leukoplakia was 3.6%, and the individual cumulative transformation rates of the dorsal tongue and ventrolateral tongue leukoplakia were 7.41% and 2.25%, respectively (Tables 1, 3), which seemed to be higher than the rate of OL of all subsites of the oral cavity in combination in previous studies. It is not possible to predict when OL will undergo malignant transformation, but it is agreeable that the longer the follow-up, the higher the rate of malignant change. ATR, which is calculated as the transformation rate divided by the time needed for OL to develop into carcinoma, could be a more scientific method to investigate the issue of malignant transformation. The time for OL to develop into carcinoma is a critical factor. If the follow-up time is short, it may not be possible to collect those cases who will transform in the future, and it is likely to underestimate the cumulative transformation rate. In a nationwide population-based retrospective cohort study of 1,898 OL patients in Taiwan, the mean time to develop oral cancer was 2.5 years [24]. A study done in the US showed that the time to the event of malignant change could be shortened because of patient selection bias in a tertiary center [25]. In the studies on OL across the globe, the mean time for malignant transformation ranged from 2 to 8.1 years [4, 25–31]. In the present study, the mean time for developing carcinoma was 3.35 years, which was consistent with previous research. The time to develop carcinoma from the dorsal tongue leukoplakia was shorter than the ventrolateral tongue leukoplakia (3.19 ± 1.94 vs. 3.51 ± 2.12 years) but the difference didn’t reach a significant difference (*P* = 0.83, Table 3). In this study, the ATR of the dorsal tongue and ventrolateral tongue leukoplakia of this study was 2.32% and 0.64%, respectively. The ATR of the published works of OL was between 1.2 and 2.9% [32–34]. The ATR of dorsal tongue leukoplakia was comparable to that reported in previous studies, but the ATR of ventrolateral tongue leukoplakia was lower. However, the differences between ATRs were not statistically significant (*P* = 0.099, Table 3). In the multivariate logistic regression analysis, the location of leukoplakia on the tongue was not a significant factor related to postoperative malignant transformation, either (Table 4). The oral tongue has been regarded as a region with a higher risk for the development of carcinoma from leukoplakia [5, 6, 35, 36]. According to the analysis in this study, the ATR of oral tongue leukoplakia in the present study did not seem to be higher than in previous studies. Considering the differences in the treatment modalities, geographical locations, cultural lifestyles, and oral and dietary habits of the patient population studied, the comparison of ATRs may not be on a comparable basis. Hence, the inherent characteristics of different studies should be considered before a conclusion is reached.

In the multivariate logistic regression analysis, pathology was the only independent prognostic factor associated with postoperative malignant transformation of oral tongue leukoplakia (OR 4.58, 95% CI 1.04–20.24, *P* = 0.045, Table 4). The pathology of OL consists of squamous hyperplasia with hyperkeratosis, and mild, moderate, and severe dysplasia. In a systemic review and meta-analysis of pooled data from 14 cohort studies, the grade of dysplasia was found to be significantly related to the development of malignant transformation [37], which was consistent with the findings in the present study. Clinicians should pay more attention and take a more aggressive attitude toward the tongue leukoplakia with higher grades of dysplasia.

Whitish patches on the tongue are usually asymptomatic but they are not easily overlooked, so delayed diagnosis does not seem to occur on the oral tongue leukoplakia, regardless of the subsites. Although dysplasia is not infrequently seen in lesions of leukoplakia, epithelial changes are still confined above the basement membrane. Although there are differences in the incidence, morphology, histological architectures, and functions between the dorsal and ventrolateral tongue, we speculate that the relatively benign nature of leukoplakia of both subsites is well subject to laser surgery so the treatment outcomes were not different. Dorsal tongue leukoplakia is not commonly seen clinicall; the reasons why the occurrence of leukoplakia on the specialized epithelium of the dorsal tongue remains an interesting and unsolved topic that needs further investigations in the future [38].

There are some limitations in this study. First, the sample size of the dorsal tongue leukoplakia was relatively small compared with that of the ventrolateral tongue leukoplakia. Large-scale, multicenter, prospective cohort studies are warranted to further investigate the disease. Second, there were some missing data in the variables due to its retrospective nature. Third, the quality histopathological diagnosis of the tissue might be more or less affected due to the thermal injury of the CO_2_ laser. Although we chose excision of the whole lesion of the tongue leukoplakia instead of vaporization, the case(s) were excluded when the pathologists could reach a consensus on the pathological diagnosis.

## Conclusions

The prevalence of the ventrolateral tongue leukoplakia was higher than that of the dorsal tongue leukoplakia. The treatment outcomes and other clinicopathological characteristics of the dorsal tongue and ventrolateral tongue leukoplakia were not different. The time for the tongue leukoplakia to develop into carcinoma for the dorsal tongue leukoplakia was shorter than that for the ventrolateral tongue leukoplakia and the ATR of the dorsal tongue leukoplakia was higher than the ventrolateral site, but these 2 factors were not statistically significant. Dorsal tongue leukoplakia is not clinically common and more studies are warranted to better understand the disease entity.

## Data Availability

Not applicable.

## Abbreviations

CO_2_: Carbon dioxide
OL: Oral leukoplakia
OR: Odds ratio
HR: Hazard ratio
CIs: Confidence intervals
CIS: Carcinoma in situ
ATR: Annual transformation rate
