# Correction to: An anatomical perspective on clinicopathological characteristics and treatment outcomes of dorsal and ventrolateral tongue leukoplakia after carbon dioxide laser surgery

**DOI:** 10.1186/s12903-021-01430-5

**Published:** 2021-02-15

**Authors:** Shih‑Wei Yang, Yun‑Shien Lee, Liang‑Che Chang, Cheng‑Han Yang, Cheng‑Ming Luo

**Affiliations:** 1 Department of Otolaryngology Head and Neck Surgery, Chang Gung Memorial Hospital, Keelung. No. 222, Mai Chin Road, Keelung, 204 Taiwan, ROC; 2 College of Medicine, Chang Gung University, Taoyuan, Taiwan, ROC; 3 New Taipei Municipal Tucheng Hospital, New Taipei City, Taiwan, ROC; 4 Genomic Medicine Research Core Laboratory, Chang Gung Memorial Hospital, Tao‑Yuan, Taiwan, ROC; 5 Department of Biotechnology, Ming Chuan University, Tao‑Yuan, Taiwan, ROC; 6 Department of Pathology, Chang Gung Memorial Hospital, Keelung, Taiwan, ROC

## Correction to: BMC Oral Health (2021) 21:45 https://doi.org/10.1186/s12903-021-01403-8

After publication of the original article [1], the authors identified an error in the Results section of the Abstract.

The incorrect sentence is: “Annual transformation rate was 4.03%”.

The correct sentence is: “Annual transformation rate was 1.08%”.

The original article has been corrected.
